# Transforming Small Ruminant Productivity Through a Farm Service Delivery Model: Evidence from a Pilot Study in Saudi Arabia

**DOI:** 10.3390/ani16071094

**Published:** 2026-04-02

**Authors:** Marimuthu Swaminathan, Khaled Aldayood, Markos Tibbo, Kakoli Ghosh, Ali Shaikhi, Nizar Haddad

**Affiliations:** 1 Food and Agriculture Organization of the United Nations (FAO), Abdul-Aziz Road, Riyadh 11421, Saudi Arabia; kakoli.ghosh@fao.org (K.G.); nizar.haddad@fao.org (N.H.); 2 Ministry of Environment, Water and Agriculture (MEWA), Eastern Ring Branch Road, Riyadh 11195, Saudi Arabia; khaalanazi@mewa.gov.sa (K.A.); ali.alshaikhi@mewa.gov.sa (A.S.); 3 Food and Agriculture Organization of the United Nations (FAO), Subregional Office for the GCC States and Yemen, Al Qala-Id Street, Abu Dhabi P.O. Box 62072, United Arab Emirates

**Keywords:** small ruminants, service delivery, livestock extension, reproductive efficiency, animal health, Saudi Arabia, economic impact, community-based interventions

## Abstract

Small ruminant production is vital for rural livelihoods and food security in Saudi Arabia but faces persistent constraints, including high feed costs, limited veterinary access, low reproductive efficiency, and weak extension services. To address these gaps, a Farm Service Delivery Model (FSDM) was piloted, which involved embedding trained livestock technicians into communities to deliver integrated on-farm services. This study evaluated the impact of the FSDM on 47 farms across three regions over 6–12 months. The key results showed significant improvements: flock size increased by 28%, the lambing rate per ewe doubled from 0.39 to 0.80, twin births tripled, mortality declined from 23.8% to 8.0%, and milk production more than doubled. Economic analysis revealed a benefit–cost ratio of 3.02, indicating high return on investment. Scaling the FSDM nationally could generate up to USD 4.8 billion in added meat and milk value over five years while reducing meat imports by 48%. The model aligns with Saudi Vision 2030 and offers a scalable and sustainable pathway to enhance productivity, resilience, and rural income. Policy recommendations include institutional integration, digital innovation, blended financing, and strengthened breeding programs.

## Simple Summary

Saudi Arabia’s small ruminant sector sustains rural livelihoods; however, it faces challenges including high feed costs, limited access to animal health services, and low reproductive efficiency. With the support of the FAO and Ministry of Environment, Water and Agriculture (MEWA), we piloted a Farm Service Delivery Model (FSDM) that embeds trained technicians in communities to provide on-farm health, feeding, and reproductive services to sheep and goat farms in Arar, Hafar al-Batin, and Jazan. Across 47 farms over a 6–12 month period, flock size increased by 28%, the number of lambs per ewe doubled, twin births tripled, mortality decreased by two-thirds, and milk output more than doubled. A benefit–cost ratio of 3.02 was obtained, indicating strong economic viability. These results demonstrate that service-based, community-embedded delivery can transform productivity and income in arid small ruminant systems and is scalable nationally through public–private partnerships and digital tools.

## 1. Introduction

Livestock production remains a cornerstone of rural economies and global food systems, providing essential nutrition, income, and sociocultural value for hundreds of millions of smallholder households [1,2]. In arid and semi-arid regions, small ruminants (sheep and goats) are particularly critical due to their adaptability to harsh climates, lower feed and water requirements than large ruminants, and their role in risk diversification [3,4]. However, despite their importance, productivity in these systems often remains suboptimal and constrained by multifaceted barriers, including inadequate access to veterinary care, poor reproductive management, high input costs, and fragmented extension services [5,6]. Closing these productivity gaps is essential for improving rural livelihoods, enhancing household resilience to climate and economic shocks, and contributing to national food security objectives [7,8].

In Saudi Arabia, recent estimates indicate that the small ruminant sector supports nearly 150,000 rural families and remains deeply embedded in local traditions and food culture, with a national flock exceeding 29 million head [9]. These figures refer to MEWA’s Statistical Report in 2023 and reflect the most recent nationally consolidated livestock census. However, the sector’s contribution to food security and rural income is limited by persistently low productivity. National performance indicators in small ruminant systems remain below those reported in well-managed intensive production environments. Evidence from Saudi Arabia shows that reproductive efficiency and lamb survival are strongly influenced by management factors such as flock size, housing systems, breeding practices, and farmer capacity, with limited use of reproductive technologies and veterinary supervision constraining productivity gains [10]. Similar challenges have been documented across arid Middle Eastern production systems, where environmental stressors, feed variability, and structural management limitations continue to affect reproductive performance and overall flock productivity [11,12]. These deficiencies result from a combination of factors, including a reliance on imported feed, which drives high production costs; limited access to quality animal health services in remote areas; a lack of structured breeding programs for indigenous breeds; and an extension apparatus historically focused on seasonal crop advising, rather than continuous livestock support [13,14].

Globally, the limitations of conventional government-centric livestock extensions have been well documented, with such models often struggling in terms of timeliness, relevance, and sustainability, particularly in remote areas [15,16]. Community-based animal health worker (CAHW) models have emerged as a successful alternative, demonstrating improved service coverage, reduced disease incidence, and enhanced trust between providers and farmers [17,18]. Furthermore, the integration of digital tools for record-keeping, decision support, and market linkage is increasingly considered vital for modernizing livestock advisory services [19,20]. Furthermore, synergies between animal health delivery and community-based breeding programs (CBBPs) have been highlighted as a pathway to simultaneously improve genetics, management, and profitability while conserving locally adapted genetic resources [21,22]. CBBPs are participatory genetic improvement programs where farmers collectively record performance, select breeding animals, and manage genetic progress within local breeds.

Recognizing these lessons and the specific needs of the livestock sector, Saudi Arabia—through its Ministry of Environment, Water, and Agriculture (MEWA) and with technical support from the Food and Agriculture Organization of the United Nations (FAO)—has designed and piloted the Farm Service Delivery Model (FSDM). This innovative model moves beyond traditional advisory approaches by embedding trained local Animal Production Technicians within farming communities to provide a bundled package of hands-on, fee-based services directly at the farm gate. Community-embedded technicians are locally recruited paraprofessionals who reside within the target communities and provide continuous, proximity-based livestock advisory and health services, thereby reducing barriers to access associated with centralized veterinary systems.

While the FSDM represents a promising pathway to improve productivity and service access, several practical and contextual limitations must be acknowledged to ensure balanced interpretation. Adoption of digital record-keeping and advisory tools—an increasingly important component of modern livestock extension—remains uneven in many smallholder settings, due to constraints relating to digital literacy, connectivity, and the ability of farmers to routinely maintain flock data [19,20]. Cost-sharing mechanisms, although central to the sustainability of service delivery, may also pose barriers for smaller or resource-constrained farms, echoing long-standing concerns regarding inequities and affordability in pluralistic and fee-based advisory systems [15,16]. Moreover, the diversity of small ruminant production systems across Saudi Arabia—from extensive rangeland systems to mixed crop–livestock systems—implies that the performance of the FSDM may vary by region due to differences in feed resource availability, breed characteristics, disease ecology, and management practices [3,4,22]. Evidence from community-based animal health and extension models also highlights that service effectiveness depends strongly on local institutional support, farmer trust, and sustained supervision—factors that, if weak, can limit long-term functionality and quality assurance [17,18]. Recognizing these potential implementation risks is essential to designing an adaptable and inclusive national FSDM-based strategy that can equitably support farms of different sizes and with varying production environments while ensuring scalability and long-term resilience.

Despite these opportunities, several context-specific challenges may hinder the broad adoption of the FSDM. Technology-enabled advisory systems may face constraints associated with digital literacy, uneven smartphone access, and variable internet connectivity in remote regions. Cost-sharing mechanisms, while central to the FSDM’s sustainability, may disadvantage smaller or low-performing farms with limited liquidity. In addition, regional differences in feed resource availability, disease ecology, and climatic stressors may produce heterogeneous outcomes across production environments. These factors highlight the importance of evaluating both the potential benefits and the operational limitations of the model.

This study presents a comprehensive evaluation of the FSDM pilot, aiming to (1) quantify the impacts of integrated service-based interventions on key productivity indicators, including flock growth, reproduction, mortality, and milk yield; (2) assess the economic viability, returns, and potential to scale-up the model; and (3) derive evidence-based policy recommendations for institutionalizing and scaling the FSDM within Saudi Arabia’s national livestock development strategy, aligning with the goals of Vision 2030 for economic diversification and food security.

## 2. Materials and Methods

### 2.1. Study Design and Pilot Implementation

The FSDM pilot was implemented across three distinct agro-ecological regions of Saudi Arabia: Arar in the Northern Borders Province, Hafar al-Batin in the Eastern Province, and Jazan in the southwestern region. These regions were purposively selected to represent the diversity of small ruminant production systems in the country, including extensive rangeland systems (Arar), mixed agro-pastoral systems (Hafar al-Batin), and smallholder mixed crop–livestock systems with relatively higher rainfall (Jazan).

A total of 50 smallholder farms were initially enrolled, of which 47 farms (20 in Arar, 15 in Jazan, and 12 in Hafar al-Batin) provided complete paired baseline and endline data and were included in the impact analysis. The intervention period lasted 12 months in Arar and 6 months in Jazan and Hafar al-Batin, covering at least one full reproductive cycle for most flocks. Outcomes were analyzed using paired before–after comparisons at the farm level; however, the unequal intervention duration across regions is acknowledged as a limitation when interpreting the results.

The study followed a quasi-experimental before–after design without a control group and was intended as a pilot evaluation to assess the feasibility, operational effectiveness, and indicative productivity impacts of the FSDM.

Given the pilot nature of the assessment, no statistical adjustment for differences in intervention duration was applied; instead, the results are analyzed as pooled before–after changes, while descriptive regional summaries are provided to contextualize variations.

### 2.2. Description of the Farm Service Delivery Model

The FSDM was designed as a demand-driven, integrated livestock service package delivered by locally recruited and trained Animal Production Technicians (APTs). Technicians underwent structured training covering animal health, nutrition, reproduction, record-keeping, and farmer advisory communication. The provided services included the following:

1. Animal identification and performance recording using ear-tagging and basic flock registers for tracking the breeding, health, and productivity of animals.
2. Feeding and husbandry advice based on locally available feed resources, in order to reduce reliance on costly imported concentrates and forages.
3. Reproduction management, including estrus detection, breeding planning, and ultrasound-based pregnancy diagnosis.
4. Preventive animal healthcare, including vaccination scheduling and strategic deworming.
5. Curative treatment and referral of complex cases to veterinary authorities.
6. Housing and welfare advice to improve shelter and enhance thermoregulation, hygiene, and animal welfare.
7. Demonstration of labor-saving equipment, such as manual milking machines, hoof clippers, and shearing equipment.

The services delivered involved scheduled farm visits and on-call support. A cost-sharing mechanism was applied, whereby farmers paid a modest service fee to enhance ownership and improve financial sustainability.

### 2.3. Baseline Diagnostic Survey

Prior to the pilot implementation, a diagnostic survey covering 84 farms across the Riyadh, Qassim, and Jazan regions was conducted to identify sector-specific constraints. The survey involved structured questionnaires administered by trained field enumerators, which collected data on production objectives, management practices, input access, and perceived challenges.

The results from this diagnostic survey were used to contextualize pilot findings, but are not included in the paired impact analysis dataset.

### 2.4. Data Collection and Quality Assurance

A mixed-methods approach was employed. Structured household surveys were administered at baseline and endline to capture demographic characteristics, management practices, and farmers’ perceptions.

Farm-level performance indicators were prospectively recorded by the APTs using standardized forms. Data quality assurance measures included

- Technician training on data recording protocols;
- Periodic supervision by FAO technical staff;
- Verification of selected farm records during monitoring visits;
- Centralized data cleaning and validation prior to analysis.

The key outcome indicators included the following.

- Flock size and composition: flock size, species (sheep versus goats), and age–sex structure.
- Reproductive performance: lambing/kidding rate, number of births, twinning rate.
- Mortality rates: number of deaths disaggregated by age.
- Production output: peak lactation milk production.
- Economic parameters: incremental income associated with livestock outputs.

A reproductive management index, reported in the baseline assessment, represented the ratio of dry females to pregnant females rather than a biological percentage indicator.

### 2.5. Statistical Analysis

Quantitative data were analyzed using Jamovi (version 2.5) and R (version 4.5.1) [23,24]. Descriptive statistics were calculated to summarize baseline characteristics and post-intervention outcomes. Impact assessment was conducted using paired sample *t*-tests comparing baseline and endline values for each farm, and statistical significance was assessed at the 5% level (*p* < 0.05). The paired *t*-tests were applied under the assumptions that (i) paired differences between baseline and endline measurements were approximately normally distributed, (ii) observations were independent at the farm level, and (iii) the before–after structure appropriately reflected the within-farm design. Normality was examined visually and using Shapiro–Wilk tests, with the normality of differences examined prior to analysis. Regional differences in selected baseline variables were assessed using Welch’s *t*-tests where heterogeneity in variance was suspected.

### 2.6. Principal Component Analysis

Principal Component Analysis (PCA) was performed to evaluate multidimensional changes in farm performance. The selected variables included lambing rate, flock growth, twinning rate, mortality rate, and milk production. Prior to analysis, all variables were standardized (z-scores) to ensure comparability. The first principal component (PC1) represented a productivity gradient, while the second component (PC2) captured mortality risk based on component loadings. Farms were classified into four performance quadrants according to the signs of the PC1 and PC2 scores: (1) productive with low mortality risk; (2) productive with higher mortality risk; (3) low productivity with high mortality risk; and (4) low productivity with low mortality risk. PC1 and PC2 had eigenvalues of 2.18 and 1.03, respectively, together explaining 64.2% of the total variance. Components with eigenvalues >1 were retained, consistent with Kaiser’s criterion. Changes in farm distribution across quadrants before and after the intervention were evaluated using chi-square tests, and Cramer’s V was calculated to estimate the effect size.

### 2.7. Economic Evaluation and Scaling Scenarios

A cost–benefit analysis was conducted from a program implementation perspective. Costs included technician salaries, training, medicines, vaccines, and logistical support, while benefits were estimated based on incremental meat and milk production attributable to improved reproduction, survival, and productivity. Inflation, seasonal price variability, and feed cost volatility were not modeled; instead, all prices were treated as constant for simplicity and to reflect a scenario-based rather than predictive modeling approach. A benefit–cost ratio (BCR) was calculated. Scaling projections were developed under simplified assumptions, including (1) proportional replication of observed productivity gains; (2) stable livestock prices and feed costs; (3) constant mortality reduction rates; and (4) unchanged production systems. Coverage scenarios of 25%, 50%, 75%, and 100% of the national flock were modeled, in order to estimate potential production gains and import substitution effects. These projections are intended as indicative scenario analyses, rather than precise forecasts.

## 3. Results

### 3.1. Baseline Characteristics of Farms and Farmers

The baseline profile revealed key insights into the structure and challenges of small ruminant production in the pilot regions. The average age of the principal farmer was 46.4 years, with a slight—though non-significant—difference between male (43.1 years) and female (48.7 years) farm managers. Households were typically large, averaging 8.4 members, and a notable pattern was the increasing involvement of younger men, suggesting a possible generational shift within the sector. Flock sizes exhibited considerable regional heterogeneity (Table 1): farms in Arar were substantially larger, averaging 406 animals (median: 341) and dominated by sheep, whereas farms in Jazan were smaller and more diversified, with an average of 170 animals (median: 156) and a more balanced sheep-to-goat ratio. This regional difference in total flock size was highly significant (Welch’s t(36.5) = −3.52, *p* = 0.001).

**Figure 1:**
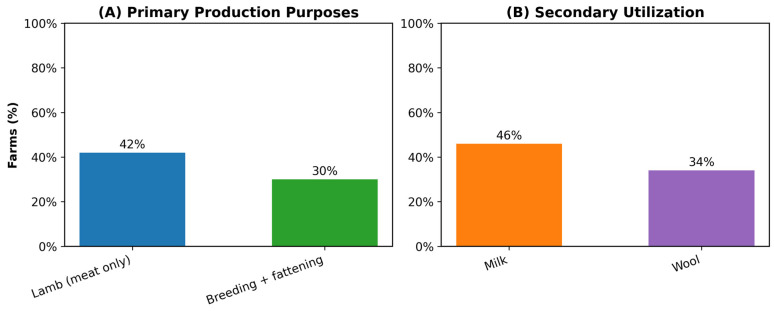
Production purposes and secondary product utilization among participating farms. Panel (**A**) presents the primary production orientation of farms, showing that 42% specialized in lamb meat production while 30% combined breeding and fattening. Panel (**B**) illustrates secondary product use, with 46% of farms reporting milk utilization and 34% reporting wool utilization; these categories are not mutually exclusive. Percentages represent the share of farms identifying each purpose or use.

Production orientation also varied (Figure 1), with 42% of farms specializing primarily in lamb/kid/buck meat production, while 30% combined breeding with fattening. Secondary use of milk (46% of farms) and wool (34%) further illustrated diversified income strategies. Pearson correlation analysis revealed a significant negative relationship between farmer age and flock size (r = −0.329, *p* = 0.038), suggesting that younger farmers tended to manage larger, potentially more commercially oriented operations. To enhance clarity and consistency, we explicitly define the production categories used in Figure 1. Here, “breeding” refers to farms focused on maintaining and reproducing core flock females and males for replacement or sale; “breeding + lamb” describes farms that pair reproduction with the fattening of offspring—including lambs, kids, or young males—for meat production; and “lamb” denotes farms specializing primarily in young stock for meat production. These definitions are applied consistently throughout the manuscript, including standardized use of the term “post-intervention” when referring to endline measurements.

Critical constraints identified in the baseline survey of 84 farms across Riyadh, Qassim, and Jazan were echoed by the pilot farmers (Table 2). The most significant challenge was the high cost and limited availability of feed, which was cited by 91% of respondents. Following this were high construction costs for shelters (76%), expensive labor (72%), and difficulties in accessing veterinary medicines and services (70%). Reproductive inefficiency was a prevalent issue, with 43% of farmers reporting low fertility rates and 48% citing problems with missed breeding opportunities. Moreover, over half of the farmers (55%) did not maintain flock records, hindering informed management and genetic selection. Baseline reproductive index analysis revealed concerning patterns, particularly in Jazan, where the proportion of dry, non-pregnant females was exceptionally high (mean: 145%), indicating severe reproductive management challenges.

To ensure correct interpretation of the baseline reproductive performance, it is necessary to clarify that the reported value of 145% does not constitute a biological fertility or lambing percentage. Instead, this figure corresponds to a diagnostic ratio representing the proportion of dry (non-pregnant) females relative to pregnant females at the time of assessment. Specifically, the indicator was calculated as follows:

$$\mathrm{Dry}-\mathrm{to}-\mathrm{Pregnant}\;\mathrm{Female}\;\mathrm{Ratio}=\frac{\mathrm{Number}\;\mathrm{of}\;\mathrm{non}-\mathrm{pregnant}\;\mathrm{females}}{\mathrm{Number}\;\mathrm{of}\;\mathrm{pregnant}\;\mathrm{females}}\times 100$$

This ratio serves as a diagnostic metric for management purposes, intended to highlight the extent of reproductive inefficiency—particularly missed breeding opportunities, suboptimal estrus detection, and delayed rebreeding—rather than to quantify biological fecundity. An index value of 145 therefore implies that the number of dry females was approximately 1.45 times the number of pregnant females at baseline, signaling substantial reproductive management challenges rather than an implausible physiological outcome.

### 3.2. Impact of FSDM Interventions on Productivity Indicators

The implementation of the FSDM resulted in statistically significant and substantial improvements across all measured productivity indicators (Table 3). The flock size exhibited a clear positive trajectory, with the average number of animals per farm increasing from 130 at baseline to 166 post-intervention (i.e., a growth rate of 28%). A paired samples *t*-test confirmed that this increase was highly unlikely to be due to chance (t(46) = −6.43, *p* < 0.001). This expansion was attributed to improved survival and active herd growth, facilitated by enhanced reproductive efficiency.

Reproductive performance exhibited the most significant change. The total number of lambs born per farm increased dramatically, rising from an average of 50.1 to 136.0, representing an increase exceeding 170%. More importantly, the lambing rate per ewe—a key efficiency metric—doubled from 0.39 to 0.80 (t(46) = −20.8, *p* < 0.001). This increase coincided with a more than three-fold rise in the number of twin births per farm, from 8.9 to 28.7 (t(46) = −11.3, *p* < 0.001). These improvements were directly linked to the bundled interventions of enhanced nutrition, strategic deworming (reducing parasitic burden on fertility) and, crucially, the implementation of ultrasound for early pregnancy diagnosis. This technology facilitated the timely re-breeding of non-pregnant ewes and improved the management of pregnant animals.

Animal health and survival outcomes markedly improved, addressing a critical baseline constraint. The average mortality rate across flocks decreased from 23.8% to 8.0% and, consequently, the number of animal deaths per farm declined from 26.4 to 11.2. Both reductions were highly significant (mortality rate: t(46) = 9.80, *p* < 0.001). This decline resulted directly from the systematic preventive health program—including vaccination and deworming—implemented by the technicians, alongside prompt treatment of sick animals.

Milk production—an important source of secondary income—demonstrated a strong positive response. The average daily milk yield per farm more than doubled, increasing from 19.8 L to 43.8 L (t(46) = −10.9, *p* < 0.001). This improvement resulted from the synergistic effects of enhanced nutrition for lactating ewes, improved health, and overall gains in reproductive performance, thereby ensuring a consistent cohort of lactating animals.

Regionally, farms in Arar showed the greatest absolute reduction in mortality, while those in Jazan exhibited stronger proportional increases in lambing rate despite a shorter intervention duration. The farms in Hafar al-Batin demonstrated intermediate improvements across most indicators, reflecting differences in baseline management capacity and production conditions.

While the pilot generated encouraging improvements across productivity, health, and economic indicators, these findings should be interpreted as proof-of-concept results rather than sector-wide estimates. The outcomes reflect the specific conditions of the 47 participating farms, the intensity of technical support provided, and the relatively short evaluation period. As such, the magnitude of observed gains may not fully translate to broader or more heterogeneous production contexts, as variability in management capacity, resource availability, adoption of recommended practices, and local ecological or market conditions could influence the degree to which similar outcomes are achievable at scale. Therefore, while these results demonstrate the potential of a community-embedded service delivery approach under pilot conditions, they do not constitute definitive evidence for its national-level impact. Further evaluations with larger samples, longer follow-up, and comparative or control-based designs will be needed to more rigorously assess its reproducibility and generalizability.

### 3.3. Multidimensional Shift in Farm Performance (PCA)

**Figure 2:**
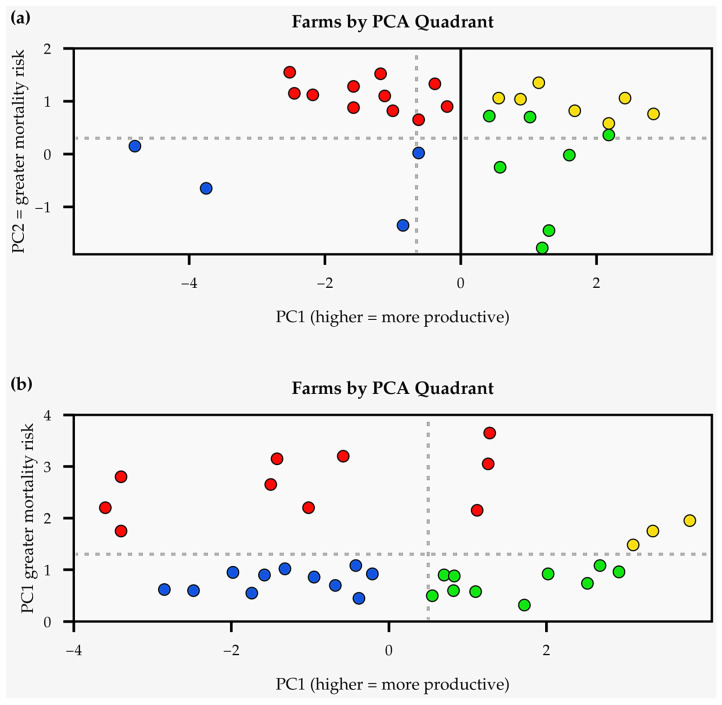
Principal Component Analysis (PCA) plots, illustrating the distribution of farms across four performance quadrants (**a**) before and (**b**) after the FSDM intervention. Quadrants are defined as follows: Q1 (Green) = Productive and Low Mortality (GOOD); Q2 (Yellow) = Productive and High Mortality (RISKY); Q3 (Red) = Low Productivity and High Mortality (POOR); and Q4 (Blue) = Low Productivity and Low Mortality (MIXED). PC1 reflects productivity level; PC2 represents mortality risk.

Principal Component Analysis provided a holistic visualization of how FSDM interventions shifted overall farm performance (Figure 2). At baseline (Figure 2a), farms were dispersed across the four quadrants, with concerning concentrations in the “risky” (productive but high mortality) and “poor” (low productivity and high mortality) categories, together comprising 66% of the farms. Following the intervention (Figure 2b), significant redistribution was observed (Table 4), with the proportion of farms in the “good” quadrant (productive and low mortality) increasing from 17% to 26%. Notably, the share of farms in the “poor” quadrant decreased dramatically from 32% to 13%, while those in the “mixed” quadrant (low productivity but low mortality) increased from 17% to 38%, suggesting a stabilization phase in which mortality was controlled before productivity fully ramped up. A chi-square test confirmed that this shift in distribution was statistically significant (χ^2^ = 9.43, *p* = 0.024), with a Cramer’s V of 0.32 indicating a medium-strength effect. This analysis underscores that the interventions led to a systemic upgrade, moving farms away from high-risk, low-output states toward more sustainable and productive configurations.

Overall, 19% of farms moved from high- to lower-risk quadrants, while 14% transitioned into the ‘productive and low-mortality’ quadrant, illustrating directional shifts consistent with improved management and health outcomes.

### 3.4. Economic and Scaling Potential Analysis

Economic evaluation of the pilot demonstrated its compelling viability (Table 5). The total cost of delivering FSDM services was compared to the value of the additional meat and milk produced. The analysis yielded a robust benefit–cost ratio (BCR) of 3.02, indicating that for every 1 Saudi Riyal (SAR) invested, approximately SAR 3.02 in benefits were generated. Projecting this model to a national scale revealed its transformative potential; in particular, under a scenario in which the FSDM is extended to 100% of the national small ruminant flock (estimated at 28.4 million heads), the five-year cumulative benefits could reach SAR 18 billion (USD 4.8 billion), representing an estimated 361,208 tons of additional meat and 709,813 tons of milk. Such an increase in domestic production could reduce Saudi Arabia’s reliance on imported meat by up to 48%, resulted in potential savings of up to SAR 4.84 billion on import bills based on 2022 volumes. At the farmer level, this scaling could translate into an average additional annual income of up to SAR 95,986 per household. Furthermore, the model demonstrates significant social returns, including the creation of an estimated 2840 skilled jobs for youth and women as Animal Production Technicians, as well as enhanced opportunities within related value chains.

## 4. Discussion

The results of the FSDM pilot indicate that a community-embedded, service-oriented model has the potential to improve key productivity and health outcomes in small ruminant systems; however, these findings should be interpreted with appropriate caution. Although marked improvements were observed across reproductive performance, mortality, and milk production, the underlying mechanisms may reflect a combination of intervention effects, context-specific factors, and short-term behavioral changes among participating farmers. The magnitude of gains—such as doubling of the lambing rate and the substantial reduction in mortality—aligns directionally with evidence from other targeted livestock management interventions, but such responses are often heterogeneous across production environments and may not be fully replicable under routine, large-scale implementations.

The observed reproductive improvements—including increases in lamb births and twinning rates—are consistent with previous studies reporting that enhanced nutrition, structured breeding management, and timely reproductive services can lead to sizeable but variable gains [25,26]. The introduction of ultrasound-based pregnancy diagnosis likely contributed to better identification of non-pregnant females and more timely rebreeding [27,28]; however, farmer adoption, technician skill level, and operational feasibility may differ at scale. Improvements of this magnitude in a pilot setting may partially reflect intensive follow-up and higher compliance than would be expected under routine field conditions. Similarly, while comparable improvements have been reported in community-based breeding and reproductive management programs in other countries [22,29], the outcomes depend heavily on the consistent application of protocols and long-term record-keeping—both of which can be challenging to maintain without sustained institutional support. However, comparisons with interventions in Ethiopia, Brazil, and India must be interpreted cautiously, as these systems differ considerably in terms of climate, feed resource availability, grazing patterns, and the structure of veterinary and extension services.

The reduction in mortality from 23.8% to 8.0% represents a particularly encouraging outcome; however, this also warrants careful interpretation. Evidence from community-based animal health worker systems indicates that a reduction in mortality is possible when preventive care and timely treatment are reliably delivered [17,30,31]. Part of the observed improvements may reflect a Hawthorne effect, whereby increased attention from technicians and researchers temporarily enhances farmer compliance and record-keeping. In the pilot, the close proximity of technicians and the structured scheduling of vaccinations and deworming likely contributed to these results. However, maintaining similar levels of service quality and responsiveness at scale may be difficult, especially in remote areas or in periods characterized by technician turnover or logistical constraints. By situating technicians within communities, the model aims to overcome the barriers to access frequently encountered with centralized veterinary services—a challenge documented in arid regions from Mongolia to the Sahel [32,33]. The reduction in mortality has profound economic implications extending beyond animal welfare; it directly increases the number of animals available for sale or breeding, stabilizes herd growth, and reduces households’ financial vulnerability to disease shocks, thereby enhancing overall resilience [34,35]. However, mortality is influenced by seasonal factors, feed conditions, and disease events, which vary across years; in this regard, the study’s 6–12 month intervention window was not sufficient to fully capture long-term variability.

The milk production gains, though notable, should also be interpreted within the context of the study’s short duration and the indirect nature of the intervention. Improvements in milk yield likely stem from general enhancements in health, nutrition, and reproductive performance rather than targeted dairy interventions, as has been shown in other smallholder systems [36,37]. Sustaining improved lactation performance requires ongoing nutritional management and careful monitoring of flock composition, which may present challenges for smaller farms facing feed cost volatility or limited access to advisory services. In Saudi Arabia, where dairy product consumption is high, incremental increases in small ruminant milk production can contribute to nutrition security and provide valuable supplemental income; particularly for women, who often manage dairy processing at the household level [38,39].

The economic results—including the estimated BCR of 3.02—provide encouraging evidence for the model’s potential financial viability; however, these projections rely on assumptions about continued performance, stable service delivery costs, and constant market conditions. Economic parameters such as feed prices, technician compensation, and consumer demand are subject to fluctuation, and these changes could influence the returns. Global evaluations of livestock interventions often show variability in the economic benefits realized over time [40,41], suggesting that caution is warranted when extrapolating pilot-level results to national scale estimates. The financial feasibility of the model appears to be strengthened by its partial cost recovery design, wherein farmers contribute service fees that offset operational expenses. Similar hybrid mechanisms have been highlighted as important for the long-term resilience of privatized or decentralized extension systems, although their effectiveness can vary depending on farmers’ capacity and local economic conditions [42,43]. The projected national-level benefits—estimated at up to USD 4.8 billion in added value and a potential reduction of approximately 48% in meat imports—should therefore be viewed as indicative rather than definitive. Nonetheless, they suggest that broader implementation of the FSDM could support key priorities of Saudi Vision 2030, including economic diversification, food security, and rural development [44]. A gradual reduction in import dependence may also help to buffer the sector against external shocks, such as global market volatility and the risk of transboundary animal disease incursions associated with live-animal imports, although the magnitude of these effects would depend on sustained program performance and broader sectoral dynamics [45,46].

The PCA plots offer a useful visualization of multidimensional performance change, suggesting that farms shifted toward more favorable productivity mortality profiles following the intervention. Nonetheless, the interpretation of these shifts is constrained by the limited duration of observation and the absence of a control group. While the movement of farms away from high-mortality quadrants is consistent with expected effects of improved service coverage, it remains possible that part of the observed variation reflects year-to-year environmental or management differences which are not directly attributable to the intervention. System-level improvements documented in other contexts [47,48] have similarly required sustained multi-year engagement to confirm the stability of observed changes [49]. These shifts should be interpreted as short-term patterns; without multi-year follow-up, the durability of these improvements cannot be confirmed.

While the pilot indicates promising pathways for scaling, the operational, institutional, and socio-economic factors that influence uptake must be acknowledged. Effective national implementation of the model depends on standardized technician accreditation, sustained supervision, farmers’ willingness to pay for services, and integration with existing veterinary and extension systems. Although digital innovations such as flock-level data platforms can improve oversight and decision making [19,50], their adoption is not uniform and may pose challenges for some producers. As noted in evaluations of similar extension and animal health delivery models [51,52], scalability often hinges on long-term financing mechanisms, clear governance arrangements, and context-specific adaptation. In addition, although linking FSDM with structured breeding programs presents significant opportunities [21,53], such efforts will require robust data systems, coordination among breeding partners, and sustained institutional commitment. In this context, investments into initiatives with strong public good characteristics, such as disease surveillance, vaccination campaigns against priority diseases, and initial digital infrastructure rollouts [54,55] are paramount.

Overall, while the FSDM pilot provides encouraging evidence of what a community embedded, service-oriented livestock support program can achieve, these findings should be viewed as indicative rather than definitive. Continued evaluation under diverse production environments, over longer time frames, and with strengthened monitoring systems will be essential to determine the consistency, scalability, and resilience of the model’s impacts.

### Limitations

While the economic projections presented in this study indicate promising potential benefits associated with the national scaling of the FSDM, these estimates should be interpreted as illustrative scenarios rather than precise forecasts. The projections necessarily rely on simplifying assumptions, including continuation of the productivity gains observed in the pilot (e.g., improved lambing rates, lower mortality, and higher milk yield), relatively stable market conditions, and the consistent availability of trained technicians and service delivery infrastructure. In practice, scaling outcomes may vary due to regional differences in production systems, fluctuations in feed prices and climatic conditions, and heterogeneity in farmers’ adoption of recommended management practices. As with many models, implementation at a broader scale may also encounter diminishing returns, resource constraints, or logistical bottlenecks that were not evident during the pilot phase. In addition, this study’s design imposed certain limitations: the sample size of 47 farms restricts generalizability, the intervention duration differed across regions, and the before–after approach without a control group limits the attribution of observed changes solely to the FSDM. Future work incorporating longitudinal monitoring, appropriate comparison groups, and formal sensitivity analyses would further strengthen our understanding of the model’s economic performance under varying assumptions and operating conditions.

## 5. Conclusions

This study demonstrated that the FSDM is a highly effective and economically viable intervention for addressing chronic productivity constraints in Saudi Arabia’s small ruminant sector. Moving from a traditional knowledge transfer extension model to a community-embedded, hands-on service delivery system, the FSDM catalyzed remarkable improvements in reproductive efficiency, animal health, and overall farm output within a short timeframe. The model’s success is rooted in its integrated approach, local ownership, and the use of appropriate technology (e.g., ultrasound) for reproduction management.

While the compelling benefit–cost ratio and significant potential for import substitution and rural job creation support national scaling of the FSDM, achieving this scale will require a deliberate policy framework that (1) institutionally anchors the FSDM within the national agricultural system, ensuring robust quality assurance; (2) accelerates digital integration to facilitate data-driven management and monitoring; (3) adopts smart, blended financing mechanisms to ensure sustainability and equity; and (4) strategically links service delivery with structured breeding programs to achieve long-term genetic gains.

In conclusion, the FSDM offers a proven and scalable blueprint for transforming small ruminant production in arid regions. Its adoption and adaptation can significantly advance Saudi Arabia’s Vision 2030 goals for food security, economic diversification, and rural prosperity, thus providing a valuable model for other countries facing similar livestock development challenges. These projections are exploratory scenario estimates, rather than forecasts, and should therefore be interpreted with appropriate caution. Achieving national coverage would also require substantial expansion and the long-term retention of trained Animal Production Technicians, which represents a non-trivial operational constraint.

## Figures and Tables

**Table 1:** Sheep and goat flock sizes managed by farmers in the Arar and Jazan regions at baseline.

Particulars	Region	Value
Number of farms—sheep only	Arar	5 farms
Number of farms—goat only	Arar	1 farm
Number of farms—sheep + goat	Jazan	12 farms
	Arar	22 farms
Average flock size—sheep + goats	Jazan	170 heads
	Arar	406 heads
Average flock size—sheep only	Jazan	130 heads
	Arar	326 heads
Average flock size—goat only	Jazan	81 heads
	Arar	1 head

**Table 2:** Major challenges faced by farmers in Riyadh, Qassim, and Jazan (*N* = 84).

No.	Challenges Faced	Number of Farmers Expressing as a Major Issue	Percentage of Farmers
1	Feed—high price and limited availability	71	91%
2	Shelter—high construction costs	59	76%
3	High labor costs	56	72%
4	Difficulties in accessing medicines and veterinary services	54	70%
5	Limited advisory services	50	74%
6	Increasing disease spread and high mortality	57	73%
7	Decreasing fertility rates in the farm	33	43%
8	High rate of missed breeding	37	48%
9	Difficulty in selecting the right breed	30	39%
10	Limited availability of quality breeding males	31	40%
11	Farm records not maintained	42	55%

**Table 3:** Changes in key productivity indicators before and after FSDM intervention (*N* = 47 farms).

Indicator	Pre-Intervention (Mean)	Post-Intervention (Mean)	Change (%)	Paired *t*-Test (*p*-Value)
Flock Size (animals/farm)	130	166	+28%	*p* < 0.001
Lamb Births (no./farm)	50.1	136.0	+171%	*p* < 0.001
Lambing Rate (lambs/ewe)	0.39	0.80	+105%	*p* < 0.001
Twin Births (no./farm)	8.9	28.7	+222%	*p* < 0.001
Mortality Rate (%)	23.8	8.0	−66%	*p* < 0.001
Milk Production (L/day/farm)	19.8	43.8	+121%	*p* < 0.001

**Table 4:** Distribution of farms across PCA performance quadrants before and after the FSDM intervention.

Quadrant	Description	No. of Farms at Start (%)	No. of Farms at End (%)
Q1	Productive and Low Mortality (GOOD)	8 (17%)	12 (26%)
Q2	Productive and High Mortality (RISKY)	16 (34%)	11 (23%)
Q3	Low Productivity and High Mortality (POOR)	15 (32%)	6 (13%)
Q4	Low Productivity and Low Mortality (MIXED)	8 (17%)	18 (38%)

**Table 5:** Projected farm production gains and investment requirements under FSDM at different national coverage levels.

Parameters	25% Population Coverage	50% Population Coverage	75% Population Coverage	100% Population Coverage
A. Farm Production Benefits				
A.1. Total stock (million heads)	7.10	14.2	21.3	28.4
A.2. Total female breeding stock (million heads)	4.97	9.94	14.91	19.88
A.3. Additional lamb production at five years (million heads)	3.71	7.42	11.13	14.84
A.5. Additional meat production at five years (tons)	90,302	180,604	270,906	361,208
A.6. Value of additional meat (million SAR)	3612	7224	10,836	14,448
A.8. Additional milk production at five years (tons)	177,453	354,907	532,360	709,813
A.9. Value of additional milk (million SAR)	887	1775	2662	3549
A.10. Total Incremental benefits (million SAR)	4499	8999	13,498	17,997
B. Investment for FSDM				
B.1. No. of Technicians required	710	1420	2130	2840
B.4. Total delivery investment costs (million SAR)	1491	2982	4473	5964
C. Economic Returns				
C. Benefit–cost ratio (BCR)	3.02	3.02	3.02	3.02
C.1. Additional income per farmer per year (SAR)	23,997	47,993	71,990	95,986
C.4. Meat import reduction (%)	12.0	24.0	36.1	48.1
C.5. Meat import reduction in value terms (billion SAR)	1.21	2.42	3.63	4.84

## Data Availability

Due to privacy agreements with participating farmers, the data are not publicly available.

## Abbreviations

The following abbreviations are used in this manuscript:

AI	Artificial Insemination
ANOVA	Analysis of Variance
BCR	Benefit–Cost Ratio
CBBP	Community-Based Breeding Program
CAHW	Community Animal Health Worker
FAO	Food and Agriculture Organization of the United Nations
FSDM	Farm Service Delivery Model
IoT	Internet of Things
MEWA	Ministry of Environment, Water and Agriculture (Saudi Arabia)
PCA	Principal Component Analysis
PPR	Peste des Petits Ruminants
PPP	Public–Private Partnership
SAR	Saudi Riyal
SRAD	Sustainable Rural Agriculture Development Program
